# DuDoCFNet: Dual-Domain Coarse-to-Fine Progressive Network for Simultaneous Denoising, Limited-View Reconstruction, and Attenuation Correction of Cardiac SPECT

**DOI:** 10.1109/TMI.2024.3385650

**Published:** 2024-09-04

**Authors:** Xiongchao Chen, Bo Zhou, Xueqi Guo, Huidong Xie, Qiong Liu, James S. Duncan, Albert J. Sinusas, Chi Liu

**Affiliations:** Department of Biomedical Engineering, Yale University, New Haven, CT 06511 USA; Department of Biomedical Engineering, Yale University, New Haven, CT 06511 USA; Department of Biomedical Engineering, Yale University, New Haven, CT 06511 USA; Department of Biomedical Engineering, Yale University, New Haven, CT 06511 USA; Department of Biomedical Engineering, Yale University, New Haven, CT 06511 USA; Department of Biomedical Engineering and the Department of Radiology and Biomedical Imaging, Yale University, New Haven, CT 06511 USA; Department of Biomedical Engineering, the Department of Radiology and Biomedical Imaging, and the Department of Internal Medicine, Yale University, New Haven, CT 06510 USA; Department of Biomedical Engineering and the Department of Radiology and Biomedical Imaging, Yale University, New Haven, CT 06511 USA

**Keywords:** Cardiac SPECT, denoising, limited-view reconstruction, attenuation correction, dual-domain learning, multi-task learning

## Abstract

Single-Photon Emission Computed Tomography (SPECT) is widely applied for the diagnosis of coronary artery diseases. Low-dose (LD) SPECT aims to minimize radiation exposure but leads to increased image noise. Limited-view (LV) SPECT, such as the latest GE MyoSPECT ES system, enables accelerated scanning and reduces hardware expenses but degrades reconstruction accuracy. Additionally, Computed Tomography (CT) is commonly used to derive attenuation maps (μ-maps) for attenuation correction (AC) of cardiac SPECT, but it will introduce additional radiation exposure and SPECT-CT misalignments. Although various methods have been developed to solely focus on LD denoising, LV reconstruction, or CT-free AC in SPECT, the solution for simultaneously addressing these tasks remains challenging and under-explored. Furthermore, it is essential to explore the potential of fusing cross-domain and cross-modality information across these interrelated tasks to further enhance the accuracy of each task. Thus, we propose a Dual-Domain Coarse-to-Fine Progressive Network (DuDoCFNet), a multi-task learning method for simultaneous LD denoising, LV reconstruction, and CT-free μ-map generation of cardiac SPECT. Paired dual-domain networks in DuDoCFNet are cascaded using a multi-layer fusion mechanism for cross-domain and cross-modality feature fusion. Two-stage progressive learning strategies are applied in both projection and image domains to achieve coarse-to-fine estimations of SPECT projections and CT-derived μ-maps. Our experiments demonstrate DuDoCFNet’s superior accuracy in estimating projections, generating μ-maps, and AC reconstructions compared to existing single- or multi-task learning methods, under various iterations and LD levels. The source code of this work is available at https://github.com/XiongchaoChen/DuDoCFNet-MultiTask.

## I. Introduction

Myocardial perfusion imaging (MPI) using Single-Photon Emission Computed Tomography (SPECT) is the most widely performed nuclear medicine exam for the diagnosis of coronary artery diseases. Reducing the injected dose can lower the potential risk of radiation to patients, but it will increase the image noise [1]. Acquiring limited-view (LV) projections using fewer solid-state detectors enables accelerated scanning and reduces hardware costs. However, it can lead to lower reconstruction accuracy due to reduced angular sampling [2]. Additionally, Computed Tomography (CT)-derived attenuation maps (μ-maps) are commonly employed for attenuation correction (AC) to improve the diagnostic accuracy of cardiac SPECT [3]. However, the extra CT scans will cause additional radiation exposure and introduce SPECT-CT misalignments [4]. Additionally, stand-alone SPECT scanners without CT assistance dominate the SPECT market share, where the conventional CT-based AC approach is not available.

Deep learning techniques have been developed to address either the low-dose (LD) denoising, LV reconstruction, or CT-free AC in nuclear medicine. Existing deep learning approaches for LD denoising in nuclear medicine are categorized into projection-domain and image-domain approaches. For the projection-domain approaches, Shiri et al. [5] applied a 2D ResNet to estimate full-dose (FD) projections from LD projections in cardiac SPECT. Similarly, Olia et al. [6] used a 2D conditional generative adversarial network (GAN) to denoise LD projections under multiple noise levels. Sun et al. [7] further used a 3D conditional GAN to denoise LD projections in cardiac SPECT. In contrast, the image-domain approaches were developed by inputting LD images into neural networks to estimate FD images [8], [9], [10], [11]. Previous studies proved that the projection-domain approaches consistently outperformed the image-domain approaches in both simulation and clinical studies [7], [12].

Previous deep learning techniques for LV reconstruction in nuclear medicine can be summarized into projection-domain, image-domain, and dual-domain methods. In the projection-domain methods, Whiteley and Gregor [13] applied a U-Net [14] to estimate full-view (FV) projections from LV projections of whole-body Positron Emission Tomography (PET). Similarly, Shiri et al. [5] used a ResNet to predict FV projections from LV projections in cardiac SPECT. In contrast, the image-domain methods were developed by feeding reconstructed LV images into neural networks to estimate FV images [15]. Amirrashedi et al. [15] further reported that the projection-domain methods outperformed the image-domain methods due to richer information in the projection presentation. Furthermore, Chen et al. [16] proposed a dual-domain method named Dual-Domain Sinogram Synthesis (DuDoSS), which utilized the image-domain output as the prior information to estimate FV projection in the projection domain. DuDoSS outperformed both projection-domain and image-domain methods [16]. Moreover, Li et al. [17] proposed a dual-domain end-to-end training method (End2End-Net) for accurate SPECT reconstruction. This method developed the image backpropagation using the true reconstructed images through image reconstruction and the dual-domain framework. In addition, deep learning techniques are also employed for the LV reconstruction of other related medical imaging modalities such as CT [18], [19], [20], [21], [22]. Specifically, a pivotal study by Wang [19] proposed to incorporate deep learning algorithms into CT image reconstruction, which demonstrated the potential of neural networks to enhance image quality based on LV CT data. Another study by Chen et al. [20] combined a deep convolutional neural network (CNN) with total variation minimization, proving that this hybrid approach can effectively improve image quality and reduce noise in LV CT reconstruction. Additionally, in a study by Yi and Babyn [21], GAN was trained to generate realistic CT images from LV data, thereby improving the structural integrity of the reconstructed CT images. Wu et al. [22] proposed a dual-domain residual-based optimization network (DRONE) for LV CT reconstruction, which consisted of three modules for embedding, refinement, and awareness respectively. It also integrates deep learning with compressed sensing for enhanced reconstruction accuracy.

Deep learning-based CT-free AC in nuclear medicine can be generally classified into indirect and direct strategies [23]. For the indirect strategy, Shi et al. [24] employed both U-Net and GAN to generate synthetic μ-maps from SPECT images, which were then utilized for the AC reconstruction of cardiac SPECT. In contrast, Yang et al. [25] applied a direct strategy in which the non-AC SPECT image was input to neural networks to estimate the AC SPECT image without the intermediate step of generating μ-maps. Furthermore, the patient non-imaging clinical information was embedded to predict more accurate AC SPECT images in a recent study [26]. Previous works proved that the indirect approaches outperformed the direct approaches in multiple clinical scanners [27], [28], [29]. However, the inaccurate estimation of the μ-map boundaries remains a major limitation of the indirect approaches as shown in [27].

While the aforementioned methods have been developed to individually tackle LD denoising, LV reconstruction, or CT-free AC in nuclear medicine, the solution for simultaneously addressing these tasks remains challenging and under-explored. Recently, multi-task simultaneous learning has been investigated in various medical imaging applications, which leverages domain-specific information across interrelated tasks to further enhance accuracy [30]. For instance, existing strategies for simultaneous registration and segmentation involved utilizing a shared encoder for multi-task feature extraction [31], [32] or employing segmentation results to assist the registration [33], [34], [35], [36], [37]. Specifically, Qin et al. [31] proposed a multi-scale network for the registration and segmentation of 2D cardiac Magnetic Resonance (MR) images. A shared encoder was applied by the segmentation and registration branches for feature extraction, leading to improved accuracy in both tasks. In contrast, Xu and Niethammer [33] trained two convolutional modules for the registration and segmentation of 3D brain MR images, respectively. The registration module was supervised by an anatomy similarity loss based on the predicted masks from the segmentation module. Another important multi-task learning application is simultaneous segmentation and classification, where the common approach is utilizing segmentation results to improve the classification accuracy [38], [39], [40], [41]. Specifically, Wu et al. [38] applied a U-Net for the segmentation and classification of lung nodules in CT. The predicted segmentation masks and bottleneck features were fused to improve the subsequent classification. In addition, Xu et al. [41] applied two cascaded convolutional modules, in which the output of the segmentation module was fed into the classification module to improve the classification of tongue images. The multi-task learning has also been explored in nuclear medicine imaging. Li et al. [42] proposed a recurrent framework for joint motion estimation and reconstruction in PET. In this study, a learned registration network was incorporated into a regularized PET image reconstruction module for simultaneous learning. Moreover, Zhou et al. [43] incorporated bidirectional LSTM layers into a Siamese pyramid network for simultaneous motion estimation and denoising in LD PET.

Although showing promising results, the above multi-task learning studies primarily focused on image-based features within single-domain frameworks. Dual-domain methods have exhibited superior performance to single-domain methods in various studies, due to augmented information constraints in both domains [16], [44], [45], [46]. Therefore, recent studies further explored fusing dual-domain features for better performance in multi-task learning [47], [48]. However, these studies were conducted based on a single imaging modality. Fusing multi-modality image features was proved to be more effective than using a single modality in deep learning applications [49] since the complementary information from multiple modalities enables more comprehensive feature representations [50]. Multi-modality information fusion have been explored in a wide range of deep learning studies based on CNN [51], [52], [53], [54], [55], [56]. Liu et al. [51] presented a Siamese convolutional network to obtain a weighted map that fuses the pixel-wise information of the two input images. Hou et al. [52] introduced a multi-modality fusion method based on a dual-channel spiking cortical model (DCSCM), which generated and combined both low- and high-frequency coefficients of images to achieve enhanced feature fusion. Xia et al. [54] proposed a fusion scheme for multi-modality medical images that utilizes and combines the image features from both the multi-scale transformation and convolutional modules. Song et al. [55] presented a cross-attention block based on non-local attention for the cross-modality fusion and registration of ultrasound and CT images. Chen et al. [56] further developed a dual-branch squeeze-fusion-excitation (DuSFE) co-attention module for cross-modality image fusion and registration of nuclear medicine image and CT. Thus, a recent study proposed a cross-domain and cross-modality network (CDI-Net) for multi-task learning [57]. However, the simple channel concatenation in CDI-Net might not effectively fuse the cross-domain or cross-modality image features. Furthermore, the basic U-Net models within the CDI-Net might not be optimal for estimating projections or μ-maps, without considering the characteristics of specific images and tasks.

Therefore, we propose a **Du**al-**Do**main **C**oarse-to-**F**ine Progressive **Net**work (DuDoCFNet) for simultaneous LD denoising, LV reconstruction, and μ-map generation of cardiac SPECT. Paired projection-domain and image-domain networks are cascaded using a multi-layer fusion (MLF) mechanism for cross-domain and cross-modality feature fusion. In the projection domain, Two-Stage Progressive Networks (TSP-Net) are utilized for LD denoising and LV restoration. The U-Net-like downsampling-upsampling framework [14] can restore general structures but might not preserve finer image details. Thus, TSP-Net utilizes a U-Net-like framework in Stage 1 to restore the coarse LV structures, followed by a non-downsampling module in Stage 2 to recover finer details of the LD projection. In the image domain, Boundary-Aware Networks (BDA-Net) enhance spatial attention on image boundaries and thus improve the boundary accuracy of the predicted μ-maps. BDA-Net first employs a shared encoder in Stage 1 to predict a coarse μ-map and its boundary image, which are then adaptively fused in Stage 2 to generate a refined μ-map. DuDoCFNet was trained end-to-end using both projection and image losses. Experiments showed that DuDoCFNet achieved superior accuracy under various iterations and LD levels.

## II. Methods

### A. Problem Formulation

The goal of this multi-task learning study is to generate the predicted FD and FV projection $\left({\hat{P}}_{FDFV}\right)$ and μ-map $\left(\hat{\mu }\right)$ with the LD and LV projection $\left(P_{LDLV}\right)$ as input, formulated as:

(1) $$\left[{\hat{P}}_{FDFV},\hat{\mu }\right]=𝓗\left(P_{LDLV}\right),$$

where $𝓗\left(\cdot \right)$ is the DuDoCFNet operator. The output labels are the ground-truth FD and FV projection $\left(P_{FDFV}\right)$ and the CT-derived μ-map $\left(\mu \right)$. Then, ${\hat{P}}_{FDFV}$ and $\hat{\mu }$ are input into a reconstruction module to output the predicted AC SPECT images. Thus, predicting ${\hat{P}}_{FDFV}$ achieves the LD denoising and LV restoration, and predicting $\hat{\mu }$ enables the CT-free AC.

### B. Data Preparation

A total of 600 anonymized clinical hybrid one-day SPECT-CT stress/rest MPI studies were included in this work. Each study along with the raw list-mode data was acquired on a GE NM/CT 570c dedicated SPECT-CT scanner following the injection of ^99m^Tc-tetrofosmin.

**Fig. 1.**
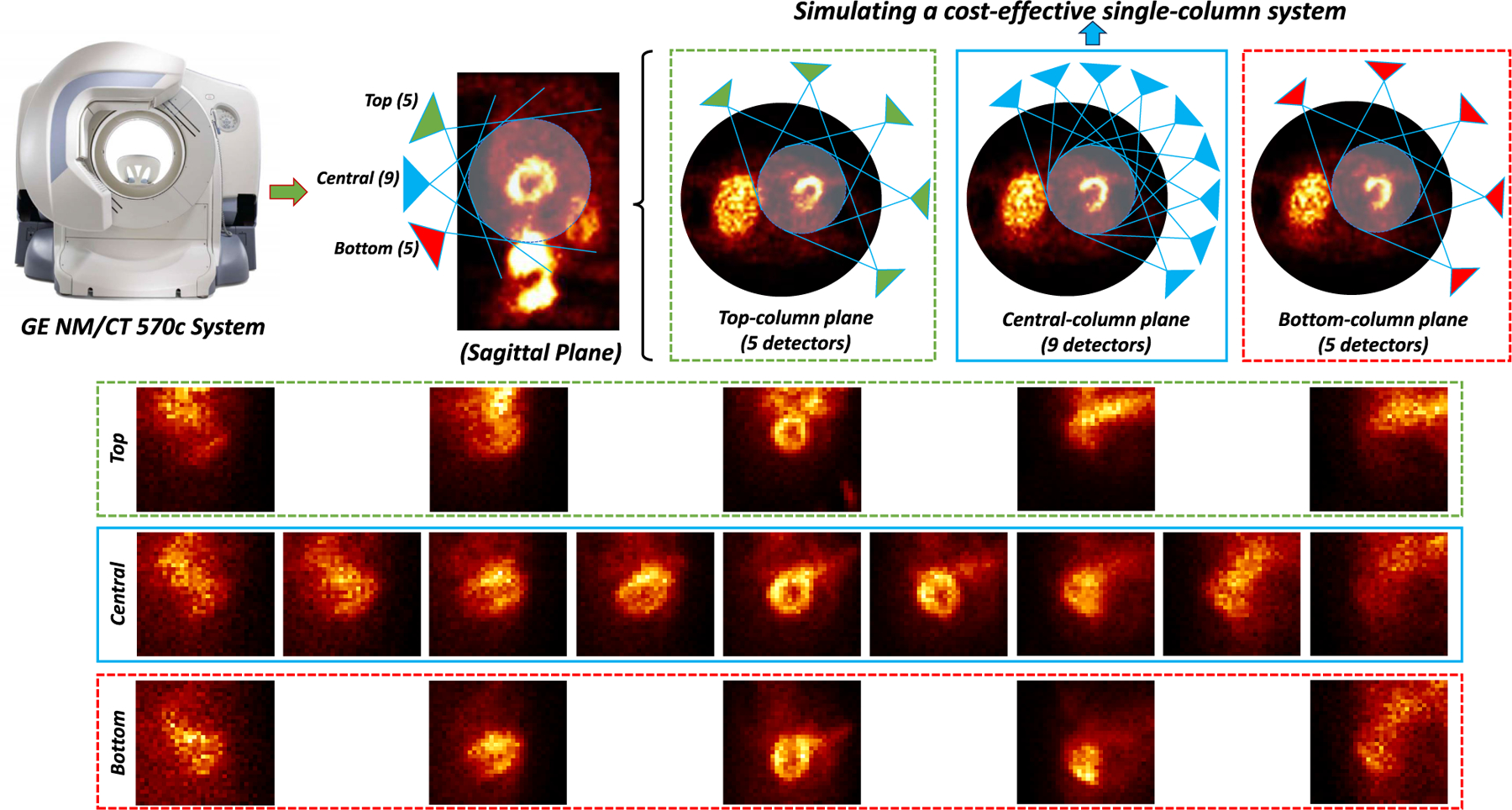
Illustration of configurations and limited-view arrangements of systems in this study. The original GE NM/CT 570c scanner has 19 detectors in total, with 5, 9, and 5 placed on the top, central, and bottom columns respectively. All the 19 detectors will collect the cardiac SPECT projection information in different spatial angles. By only including the central 9 detectors, we simulated a cost-effective single-column scanner, such as the GE MyoSPECT ES system, as denoted in the blue solid box above.

The GE NM/CT 570c scanner features a cylindrical surface array of 19 detectors, organized into three columns. Specifically, there are 5, 9, and 5 detectors placed on the top, central, and bottom columns, respectively [58]. These detectors collectively focus on the central cardiac regions to gather photon projection data from various spatial angles. The GE MyoSPECT ES scanner is a latest cost-effective variant of the GE NM/CT 570c scanner, which only comprises the 9 central-column detectors. By only including the central 9 detectors, we simulated the configurations of the GE MyoSPECT ES scanner, as presented in Fig. 1.

The LV projections is generated by zero-padding the top and bottom columns of the FV projections while retaining the central-column projections unchanged. Replicated padding was utilized to expand all 19-layer projections to 20 layers, facilitating the convolutional and downsampling computations. The LD projection was generated by randomly decimating the FD list-mode data with a default 10% downsampling rate. $P_{LDLV}\in {\mathbb{R}}^{32\times 32\times 20}$ was produced by conducting both the LV and LD downsampling. $P_{FDFV}\in {\mathbb{R}}^{32\times 32\times 20}$ was the original FD and FV projection. The CT-derived μ-maps $\left(\mu \in {\mathbb{R}}^{72\times 72\times 40}\right)$ with a voxel size of 4 × 4 × 4 mm^3^ were well registered and resolution-matched to the SPECT images. 250, 100, and 250 cases were utilized for training, validation, and testing.

### C. DuDoCFNet Overview

**Fig. 2.**
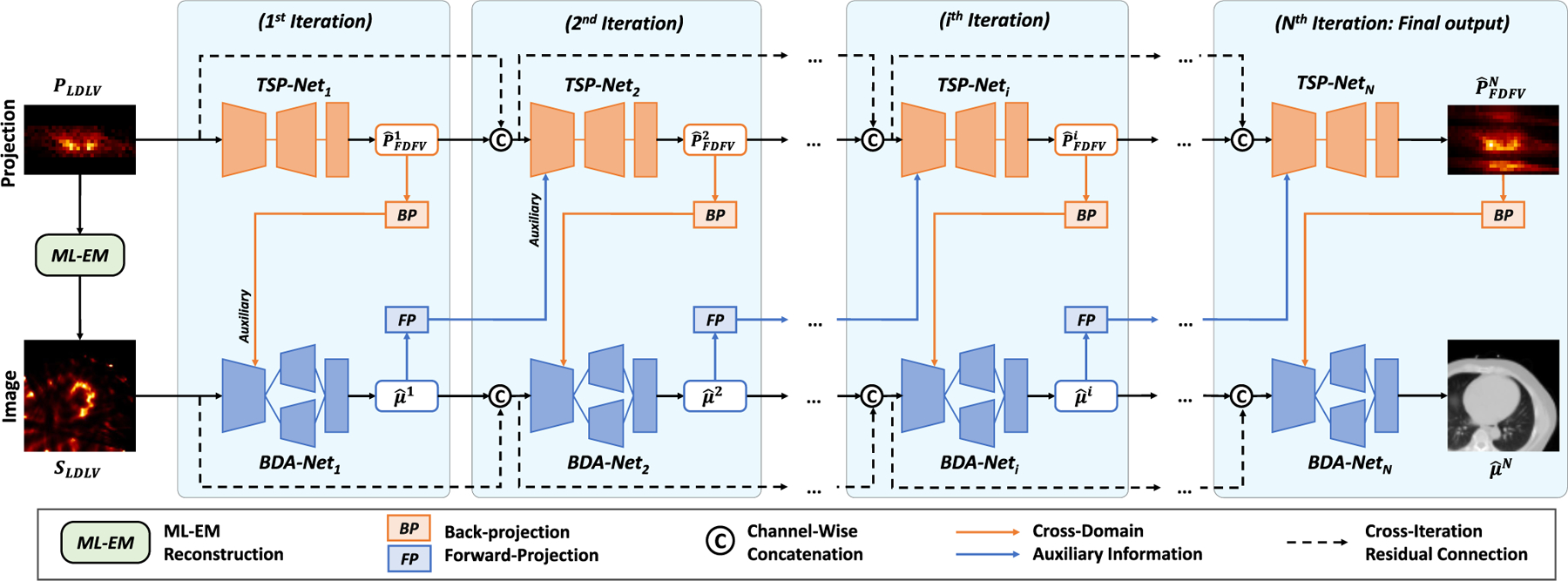
Overview of the **Du**al-**Do**main **C**oarse-To-**F**ine Progressive **Net**work (DuDoCFNet). In each iteration, DuDoCFNet employs a Two-Stage Progressive Network (TSP-Net) in the projection domain for denoising and restoration of the LD and LV projections, and a Boundary-Aware Network (BDA-Net) in the image domain for predicting μ-maps. All the TSP-Nets and BDA-Nets are cascaded to enable cross-domain and cross-modality feature fusion. The predicted projection and μ-map of the last iteration are employed as the final prediction outputs of DuDoCFNet.

**Fig. 3.**
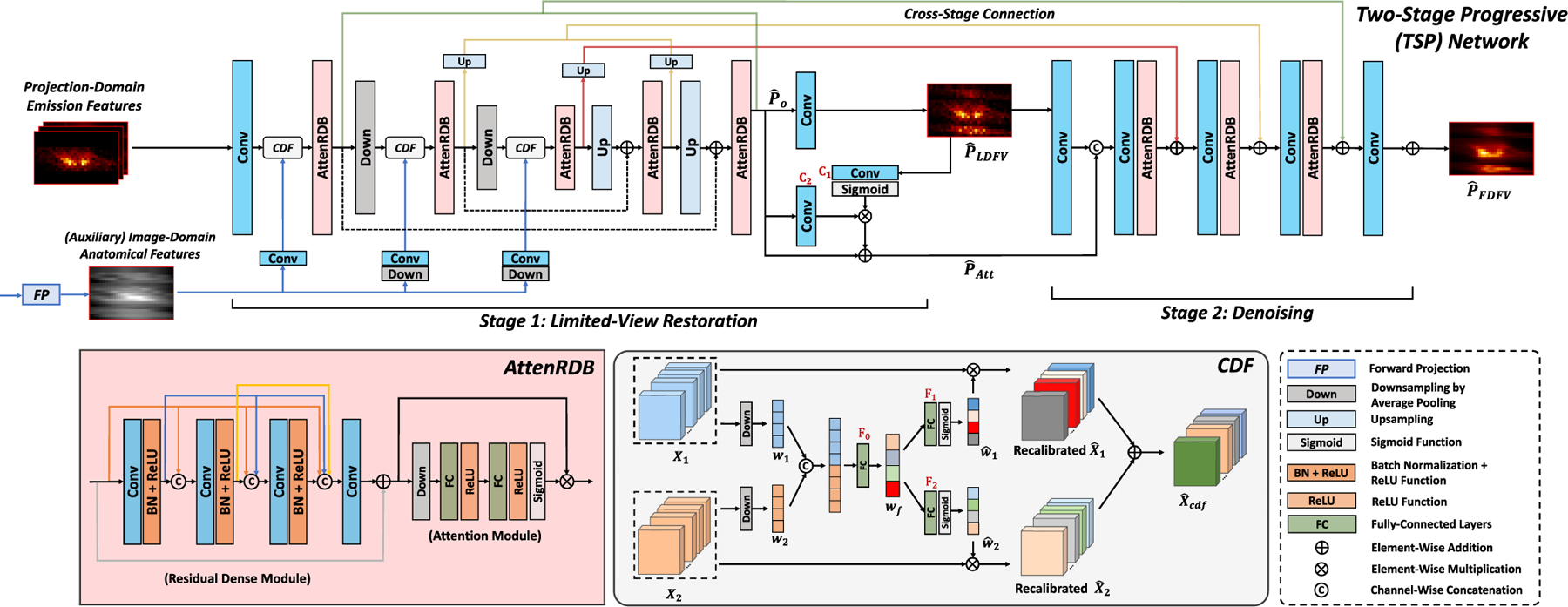
Two-Stage Progressive Network (TSP-Net). In Stage 1, a U-Net-like structure is utilized to achieve the LV restoration. The auxiliary anatomical features are fed into multiple downsampling layers as the multi-layer fusion (MLF) mechanism. Cross-Domain Feature Fusion (CDF) modules recalibrate the channel weights for adaptive feature fusion. A non-downsampling module is employed in Stage 2 for the LD denoising.

**Fig. 4.**
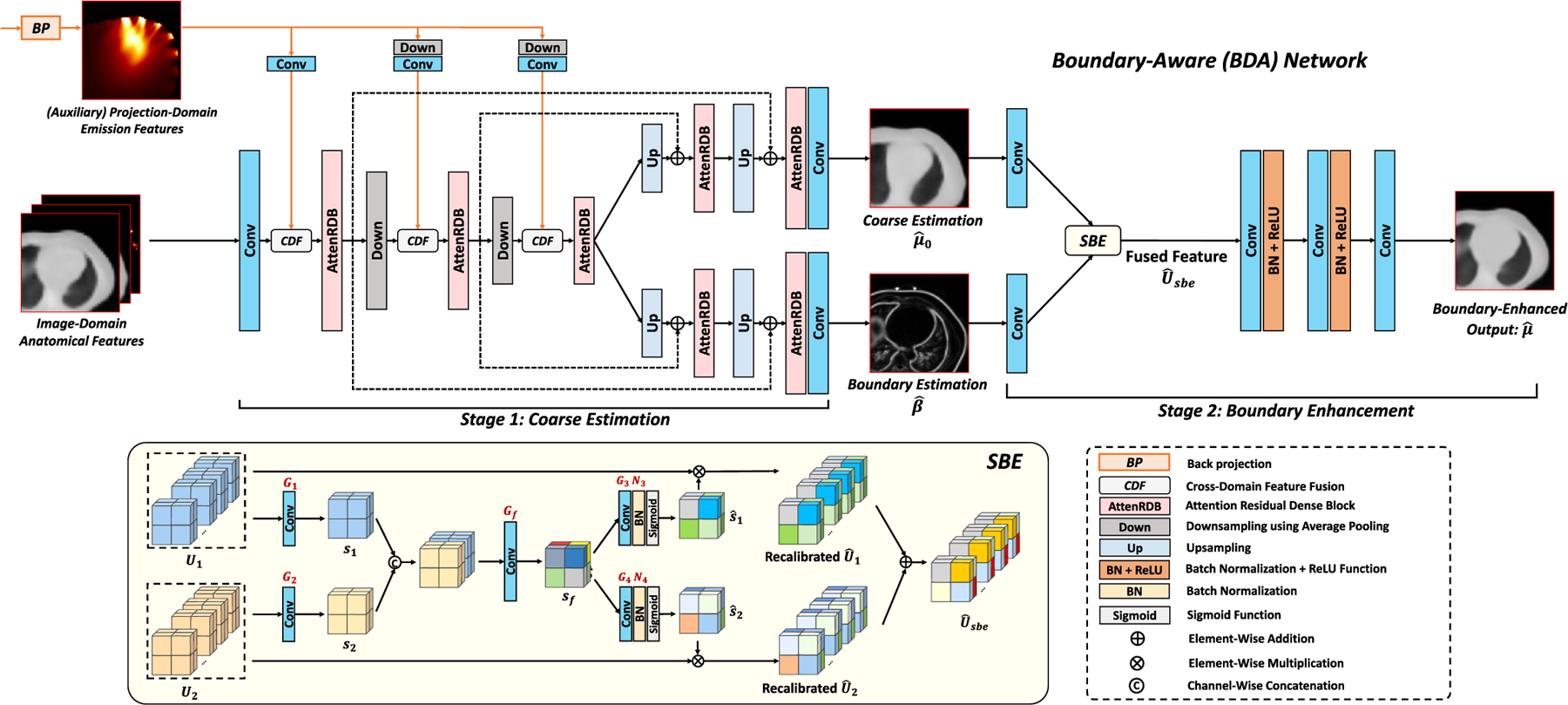
Boundary-Aware Network (BDA-Net). A shared encoder and two task-specific decoders are utilized to estimate a coarse μ-map and its boundary image. Cross-domain features are embedded in multiple downsampling layers as the multi-level fusion. The estimated μ-map and boundary image are jointly fed into a Spatial Boundary Enhancement (SBE) module to enhance the boundary accuracy of the final refined μ-map.

The framework of DuDoCFNet is presented in Fig. 2. $P_{LDLV}\in {\mathbb{R}}^{32\times 32\times 20}$ is first input to a Maximum-Likelihood Expectation-Maximization module (ML-EM, 30 iterations), and then generates the output LD and LV SPECT image $S_{LDLV}\in {\mathbb{R}}^{72\times 72\times 40}$. Then, $P_{LDLV}$ and $S_{LDLV}$ are fed into DuDoCFNet to simultaneously estimate $P_{FDFV}$ and μ. The projection-domain *TSP-Nets* (Fig. 3) and image-domain *BDA-Nets* (Fig. 4) are cascaded for cross-domain and cross-modality feature fusion. The details of *TSP-Net* and *BDA-Net* are described in subsequent subsections.

In the 1^*st*^ iteration, $P_{LDLV}$ is first input into *TSP-Net*_1_ and output the predicted FD and FV projection ${\hat{P}}_{FDFV}^{1}$. After Back-Projection (BP), ${\hat{P}}_{FDFV}^{1}\in {\mathbb{R}}^{32\times 32\times 20}$ is input to *BDA-Net*_1_ and turns into an image-domain feature map with a matrix size of 72×72×40, leveraging the auxiliary emission information to enhance the μ-map estimation. $S_{LDLV}$ is also input into *BDA-Net*_1_ to provide the image-domain information. An MLF mechanism (to be described later) is employed in *BDA-Net*_1_ to adaptively fuse the cross-domain features to produce a more accurate ${\hat{\mu }}^{1}$.

Then, in the $i^{th}\left(i\geq 2\right)$ iteration, the image-domain output of the previous iteration, ${\hat{\mu }}^{\left(i-1\right)}$, is input to *TSP-Net*_*i*_ after Forward-Projection (FP). The FP operator turns ${\hat{\mu }}^{\left(i-1\right)}\in {\mathbb{R}}^{72\times 72\times 40}$ into a projection-domain feature map with a matrix size of 32 × 32 × 20, employing the auxiliary anatomical information of ${\hat{\mu }}^{\left(i-1\right)}$ to assist the projection estimation. Additionally, the predicted projections from previous $\left(i-1\right)$ iterations are concatenated and input into *TSP-Net*_*i*_, preserving the previously estimated emission features to gradually enhance the prediction accuracy. *TSP-Net*_*i*_ also utilizes the MLF mechanism to adaptively fuse the cross-domain and cross-modality features. The output of *TSP-Net*_*i*_ is formulated as:

(2) $$\begin{array}{l} {\hat{P}}_{FDFV}^{i}\\ ={𝓣}_{i}\left(\Gamma_{f}\left({\hat{\mu }}^{\left(i-1\right)}\right),\left\{{\hat{P}}_{FDFV}^{1},\cdots ,{\hat{P}}_{FDFV}^{\left(i-1\right)},P_{LDLV}\right\}\right), \end{array}$$

where ${𝓣}_{i}$ is the *TSP-Net*_*i*_ and $\Gamma_{f}$ is the FP operator. $\left\{\cdot \right\}$ refers to channel-wise concatenation. Similarly, ${\hat{P}}_{FDFV}^{i}$ after BP is input to *BDA-Net*_*i*_, employing the auxiliary emission information of ${\hat{P}}_{FDFV}^{i}$ to assist the μ-map estimation. The predicted μ-maps from previous $\left(i-1\right)$ iterations are also concatenated and input into *BDA-Net*_*i*_, generating ${\hat{\mu }}^{i}$ as:

(3) $${\hat{\mu }}^{i}={𝓓}_{i}\left(\Gamma_{b}\left({\hat{P}}_{FDFV}^{i}\right),\left\{{\hat{\mu }}^{1},\cdots ,{\hat{\mu }}^{\left(i-1\right)},S_{LDLV}\right\}\right),$$

where ${𝓓}_{i}$ is the *BDA-Net*_*i*_ and $\Gamma_{b}$ is the BP operator. The predictions of the $N^{th}$ iteration (N represents the total number of iterations, default 5), ${\hat{P}}_{FDFV}^{N}$ and ${\hat{\mu }}^{N}$, are the prediction outputs of DuDoCFNet as described in Eq. 1. The predicted projection ${\hat{P}}_{FDFV}^{N}\in {\mathbb{R}}^{32\times 32\times 20}$ and μ-map ${\hat{\mu }}^{N}\in {\mathbb{R}}^{72\times 72\times 40}$ are then input to an offline ML-EM reconstruction module (30 iterations) to generate the final reconstructed AC SPECT image ${\hat{S}}_{FDFV}\in {\mathbb{R}}^{72\times 72\times 40}$.

### D. TSP-Net in Projection Domain

The architecture of TSP-Net is illustrated in Fig. 3. As mentioned above, the downsampling-upsampling framework can recover general structures but not finer image details. Thus, TSP-Net uses a U-Net-like framework for coarse LV restoration in Stage 1 and a non-downsampling module for finer LD denoising in Stage 2. As described in Eq. 2, the inputs to TSP-Net include the projection-domain emission features and the auxiliary image-domain anatomical features after FP.

In Stage 1, residual dense blocks with attention (AttenRDB, depicted at the bottom left of Fig. 3) are employed for image feature extractions in TSP-Net. The emission and the auxiliary anatomical features are connected at multiple downsampling layers to enable effective feature fusion in various spatial dimensions, which is the MLF mechanism. Due to the discrepancy between the two imaging modalities, we propose a Cross-Domain Feature Fusion (CDF) module to calibrate the channel-wise weights before fusing the two-modality features. As shown at the bottom right of Fig. 3, the two inputs of CDF, $X_{1}$ and $X_{2}\in {\mathbb{R}}^{h\times w\times d\times c}$ (h, w, d, and c refer to height, width, depth, and number of channels), are first downsampled into $w_{1}$ and $w_{2}\in {\mathbb{R}}^{c}$ to encode the channel-wise weight features. Then, $w_{1}$ and $w_{2}$ are fused into $w_{f}\in {\mathbb{R}}^{2c}$ using concatenation and a fully-connected layer, described as:

(4) $$w_{f}=F_{0}\left(\left\{D\left(X_{1}\right),\;\;\;D\left(X_{2}\right)\right\}\right),$$

where $D\left(\cdot \right)$ is downsampling by average pooling and $F_{0}$ is the fully-connected layer. Then, $w_{f}$ is utilized to generate the recalibration weights ${\hat{w}}_{1}$ and ${\hat{w}}_{2}\in {\mathbb{R}}^{c}$, which are applied back to $X_{1}$ and $X_{2}$ using element-wise multiplication. The recalibrated features are then concatenated to generate the CDF output ${\hat{X}}_{cdf}\in {\mathbb{R}}^{h\times w\times d\times 2c}$, formulated as:

(5) $${\hat{X}}_{cdf}=\left\{\sigma \left(F_{1}\left(w_{f}\right)\right)⊗X_{1},\sigma \left(F_{2}\left(w_{f}\right)\right)⊗X_{2}\right\},$$

where $\sigma \left(\cdot \right)$ is the sigmoid activation function and ⊗ refers to the element-wise multiplication. Therefore, CDF effectively fuses the channel information and optimizes the fusion weights of the anatomical and emission features. Next, the output of the last decoding layer, ${\hat{P}}_{0}$, goes through a 1×1×1 convolution layer to generate the predicted LD and FV projection ${\hat{P}}_{LDFV}$.

In Stage 2, ${\hat{P}}_{LDFV}$ is input to a non-downsampling module for denoising. Meanwhile, we apply a self-attention block to refine and improve the ${\hat{P}}_{LDFV}$ before feeding it to the next stage, inspired by [61]. The self-attention output is:

(6) $${\hat{P}}_{Att}={\hat{P}}_{0}+C_{2}\left({\hat{P}}_{0}\right)⊗\sigma \left(C_{1}\left({\hat{P}}_{LDFV}\right)\right),$$

where $C_{1}\left(\cdot \right)$ and $C_{2}\left(\cdot \right)$ are 1 × 1 × 1 convolution. ${\hat{P}}_{Att}$ is fed to Stage 2 for feature reserving. In addition, as depicted at the top of Fig. 3, the image features from multiple layers of Stage 1 are concatenated and input into consecutive AttenRDB layers in Stage 2. This utilizes the information from Stage 1 in multiple spatial dimensions to gradually enhance prediction accuracy in Stage 2. Finally, ${\hat{P}}_{FDFV}$ is generated as the output of Stage 2. The training loss of *TSP-Net*_*i*_ is calculated based on its outputs from both stages, formulated as:

(7) $${𝓛}_{Proj}^{i}={\left\|{\hat{P}}_{LDFV}^{i}-P_{LDFV}\right\|}_{1}+{\left\|{\hat{P}}_{FDFV}^{i}-P_{FDFV}\right\|}_{1},$$

where ${\hat{P}}_{LDFV}^{i}$ and ${\hat{P}}_{FDFV}^{i}$ indicate the predictions in the $i^{th}$ iteration. $P_{LDFV}$ is the ground-truth LD and FV projection.

### E. BDA-Net in Image Domain

The structure of BDA-Net is shown in Fig. 4. As mentioned above, the inaccurate boundary estimation poses a significant limitation in predicting μ-maps [27]. To address this, BDA-Net first uses a shared encoder in Stage 1 to predict a coarse μ-map and a boundary image as the preliminary results. Then, the predicted coarse μ-map and boundary image are fused in Stage 2 to enhance the spatial attention on the image boundaries, thus generating a refined μ-map. As described in Eq. 3, the inputs to BDA-Net include the image-domain anatomical features and the auxiliary projection-domain emission features after BP.

In the shared encoder of Stage 1, the MLF mechanism is utilized to connect and fuse the anatomical and the auxiliary emission features at multiple downsampling layers. Then, inspired by [32], two task-specific decoders are used to predict a coarse image ${\hat{\mu }}_{0}$ and a boundary profile $\hat{\beta }$. Next, a Spatial Boundary Enhancement (SBE) module utilizes ${\hat{\mu }}_{0}$ and $\hat{\beta }$ as inputs to fuse the spatial features and enhance the spatial attention on the image boundaries. As shown at the bottom of Fig. 4, the input $U_{1}$ and $U_{2}\in {\mathbb{R}}^{72\times 72\times 40\times c}$ are squeezed into $s_{1}$ and $s_{2}\in {\mathbb{R}}^{72\times 72\times 40\times 1}$ using 3 × 3 × 3 convolutions. Then, $s_{1}$ and $s_{2}$ are fused by concatenation and a convolutional layer, generating the combined spatial feature $s_{f}\in {\mathbb{R}}^{72\times 72\times 40\times 2}$ as:

(8) $$s_{f}=G_{f}\left(\left\{G_{1}\left(U_{1}\right),\;\;\;G_{2}\left(U_{2}\right)\right\}\right),$$

where $G_{1}\left(\cdot \right)$, $G_{2}\left(\cdot \right)$, $G_{f}\left(\cdot \right)$ refer to the 3×3×3 convolutions. Then, ${\hat{s}}_{1}$ and ${\hat{s}}_{2}$ are generated from $s_{f}$ and applied back to $U_{1}$ and $U_{2}$ using element-wise multiplication. Then, the recalibrated spatial features are concatenated to generate the SBE output ${\hat{U}}_{sbe}\in {\mathbb{R}}^{72\times 72\times 40\times 2c}$, formulated as:

(9) $${\hat{U}}_{sbe}=\left\{\sigma \left(N_{3}\left(G_{3}\left(s_{f}\right)\right)\right)⊗U_{1},\;\;\;\sigma \left(N_{4}\left(G_{4}\left(s_{f}\right)\right)\right)⊗U_{2}\right\},$$

where $G_{3}\left(\cdot \right)$ and $G_{4}\left(\cdot \right)$ refer to convolutions. $N_{3}\left(\cdot \right)$ and $N_{4}\left(\cdot \right)$ are the batch normalization layers. Then, ${\hat{U}}_{sbe}$ is input to the subsequent convolutional module in Stage 2 to extract the boundary information. Finally, a refined μ-map with more accurate boundaries, $\hat{\mu }$, is generated as the output of BDA-Net. The training loss of *BDA-Net*_*i*_ is calculated based on its preliminary and refined outputs in both stages, formulated as:

(9) $${𝓛}_{Img}^{i}={\left\|{\hat{\beta }}^{i}-\beta \right\|}_{1}+{\left\|{\hat{\mu }}_{0}^{i}-\mu \right\|}_{1}+{\left\|{\hat{\mu }}^{i}-\mu \right\|}_{1},$$

where ${\hat{\beta }}^{i}$, ${\hat{\mu }}_{0}^{i}$, and ${\hat{\mu }}^{i}$ are predicted boundary and μ-maps in the $i^{th}$ iteration. β represents the boundary of μ and is determined by calculating the 3D gradients of μ, formulated as:

(11) $$\beta ={\left\|\nabla \mu_{x}\right\|}_{2}+{\left\|\nabla \mu_{y}\right\|}_{2}+{\left\|\nabla \mu_{z}\right\|}_{2}.$$

### F. Implementation Details and Ablation Studies

DuDoCFNet is end-to-end trained using both projection and image losses in all iterations. The overall loss function is:

(12) $$𝓛=\sum_{i=1}^{N}\left(\alpha_{P}\;\;{𝓛}_{Proj}^{i}+\alpha_{I}\;\;{𝓛}_{Img}^{i}\right),$$

where N is the total number of iterations (default: 5). ${𝓛}_{Proj}^{i}$ and ${𝓛}_{Img}^{i}$ are the projection and image losses described in Eq. 7 and 10. $\alpha_{P}$ and $\alpha_{I}$ are loss weights. DuDoCFNet achieves the optimal performance when $\alpha_{P}=1$ and $\alpha_{I}=0.2$.

In this study, DuDoCFNet was compared to various single- and multi-task learning methods. For estimating projections, U-Net [14], Densely-Connected U-Net (DenseUNet) [59], and Attention U-Net (AttenUNet) [60] with squeeze-excitation mechanism [62] were employed to estimate $P_{FDFV}$ using $P_{LDLV}$ as the input. The aforementioned networks were utilized twice in sequence to first perform denoising and then LV reconstruction, generating the final predicted $P_{FDFV}$ as the output. DuDoSS [16], the dual-domain data consistency prediction method, was included as the previous state-of-the-art method for limit-view reconstruction and denoising in SPECT imaging. For the μ-map estimation, U-Net, DenseUNet, and AttenUNet were utilized to predict μ using $S_{LDLV}$ as the input. The multi-energy-window image generation approach using AttenUNet with squeeze-excitation attention mechanism [62], was the previous state-of-the-art method for μ-map generation in dedicated cardiac SPECT [27]. Moreover, the multi-task learning method, CDI-Net [57], was applied to simultaneously estimate $P_{FDFV}$ and μ. CDI-Net is set to run for 5 iterations as in [57].

Ablation studies of DuDoCFNet were also conducted for further evaluation. Either TSP-Net or BDA-Net was solely used to predict $P_{FDFV}$ or μ, to assess the impact of the multi-task learning framework on the prediction accuracy. Furthermore, to assess the effect of the progressive learning strategy, Stage 2 of TSP-Net or BDA-Net in DuDoCFNet is removed to produce DuDoCFNet w/o TSP and DuDoCFNet w/o BDA. Moreover, the MLF mechanism is replaced by input-channel concatenations as in [57] to produce DuDoCFNet w/o MLF.

**Fig. 5.**
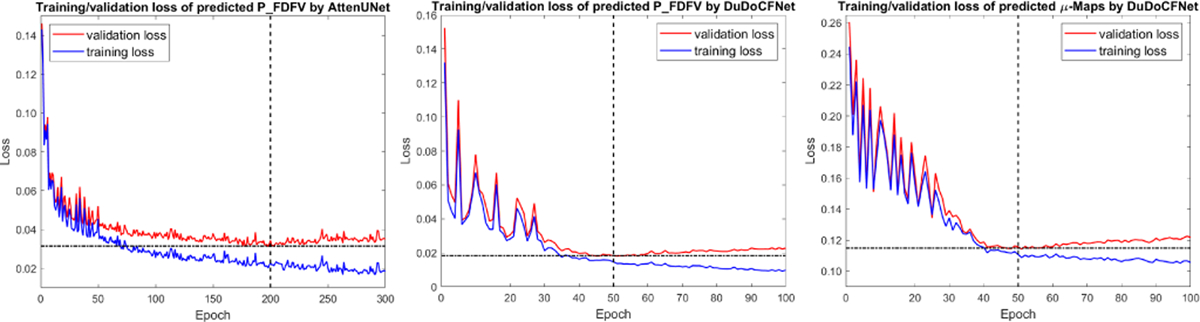
The training and validation losses of a single-task learning group (AttenUNet) and a multi-task simultaneous learning group (DuDoCFNet). The left figure shows the losses of the predicted FDFV projections by AttenUNet. The middle and right figures show the losses of the simultaneously predicted FDFV projections and μ-maps by DuDoCFNet. The single-task learning network reaches overfitting and convergence at about 50 epochs. In contrast, the multi-task learning network reaches overfitting and convergence at about 200 epochs.

All the aforementioned frameworks were developed using PyTorch [63] with Adam optimizers ($\beta_{1}=0.5$, $\beta_{2}=0.999$). The projection-domain networks for predicting FDFV projections, including the projection-domain U-Net, DenseUNet, AttenUNet, and TSP-Net, were trained with a learning rate of 1 × 10^−3^ and a batch size of 2. The image-domain networks for predicting μ-maps, including the image-domain U-Net, AttenUNet, and BDA-Net were trained with a learning rate of 1 × 10^−4^ and a batch size of 2. For the dual-domain methods, including DuDoSS, End2End-Net, CDI-Net, and DuDoCFNet, the projection- and image-domain sections were trained with learning rates of 1×10^−3^ and 1×10^−4^ respectively, and a batch size of 2. Batch normalization was implemented to stabilize the learning process by re-centering and re-scaling the inputs from the previous layer [64]. A learning rate decay policy with a step size of 1 and a decay rate of 0.99 was employed to avoid overfittings [65]. The single-task learning networks were trained for 200 epochs, while the multi-task simultaneous learning networks including CDI-Net and DuDoCFNet were trained for 50 epochs to reach overfitting and convergence, as shown in Fig. 5. The training parameters were tuned by inspecting the performance on the validation datasets with repeated and comparative experiments. DuDoCFNet is designed to be super light-weighted by reducing the number of convolutional channels from 32 to 16 and reducing the number of convolutional layers in each densely-connected module from 6 to 4, to minimize the computational costs of the iterative framework.

## III. Results

### A. Predicted Projections

Normalized Mean Square Error (NMSE), Structural Similarity (SSIM), and Peak Signal-to-Noise Ratio (PSNR) are used for voxel-wise quantitative evaluations of predicted projections, μ-maps, and reconstructed AC images. Clinical 17-segment polar maps are generated from AC images and quantified using Absolute Percent Error (APE) [26].

**Fig. 6.**
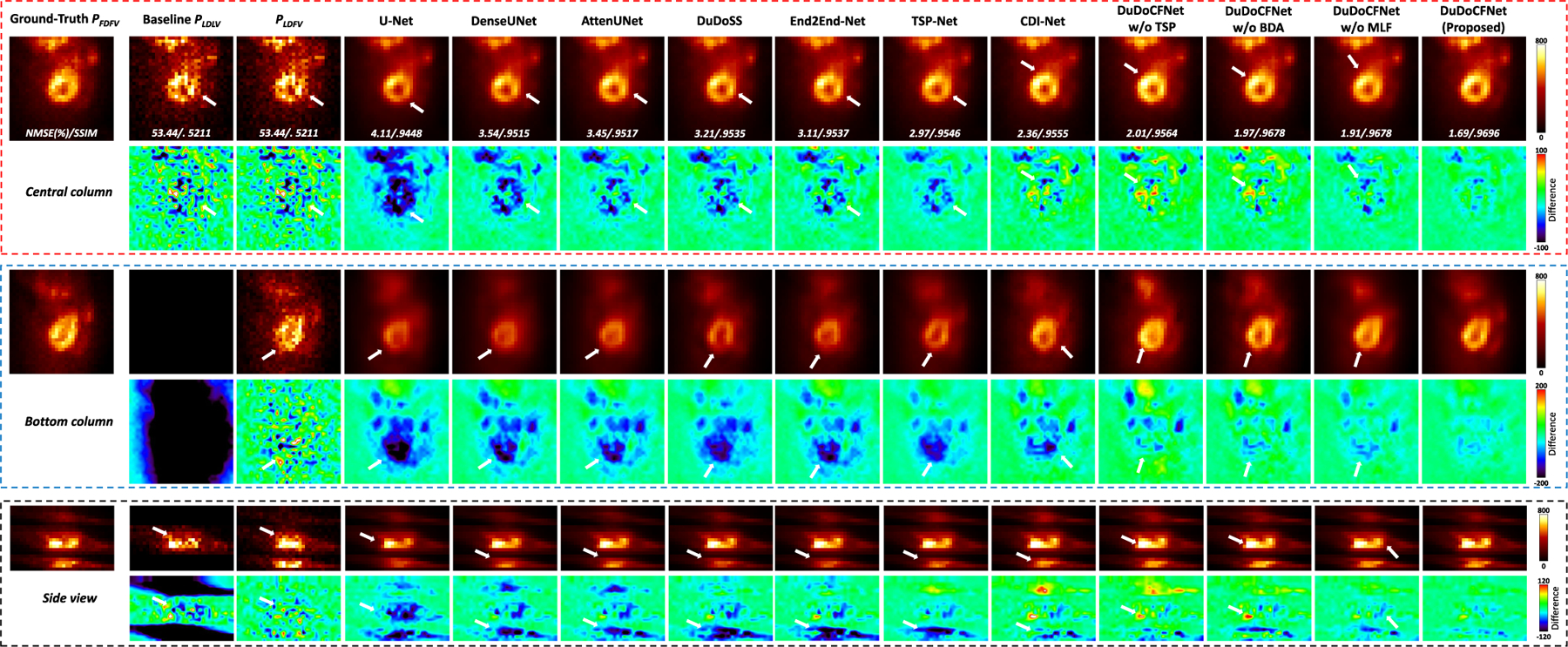
Predicted FD and FV projections displayed in the central-column angle, bottom-column angle, and side view. White arrows denote the regions with over- or under-estimated projection intensities. NMSE and SSIM between the predicted and ground-truth projections are annotated.

Fig. 6 shows the predicted FD and FV projections. We can observe that the single-task learning methods including U-Net, DenseUNet, AttenUNet, DuDoSS, and End2End-Net significantly underestimate the projection intensities in the cardiac regions. In contrast, TSP-Net outputs more accurate projections than AttenUNet, showing the effectiveness of the progressive learning framework (Fig. 3) in recovering the FD and FV projections. The multi-task learning method, CDI-Net, produces more accurate projections than single-task learning methods. Moreover, DuDoCFNet outperforms CDI-Net and ablation groups, confirming the efficacy of our proposed progressive learning strategies and the MLF mechanism in enhancing estimation accuracy. Table I lists the quantitative evaluations of the predicted projections. It can be observed that TSP-Net shows higher accuracy than other single-task learning methods but uses fewer parameters. DuDoCFNet exhibits higher accuracy than ablation study groups and CDI-Net (NMSE, 1.83% vs 2.28%, p*<*0.001) but has fewer parameters than CDI-Net.

### B. Predicted Attenuation Maps

**Fig. 7.**
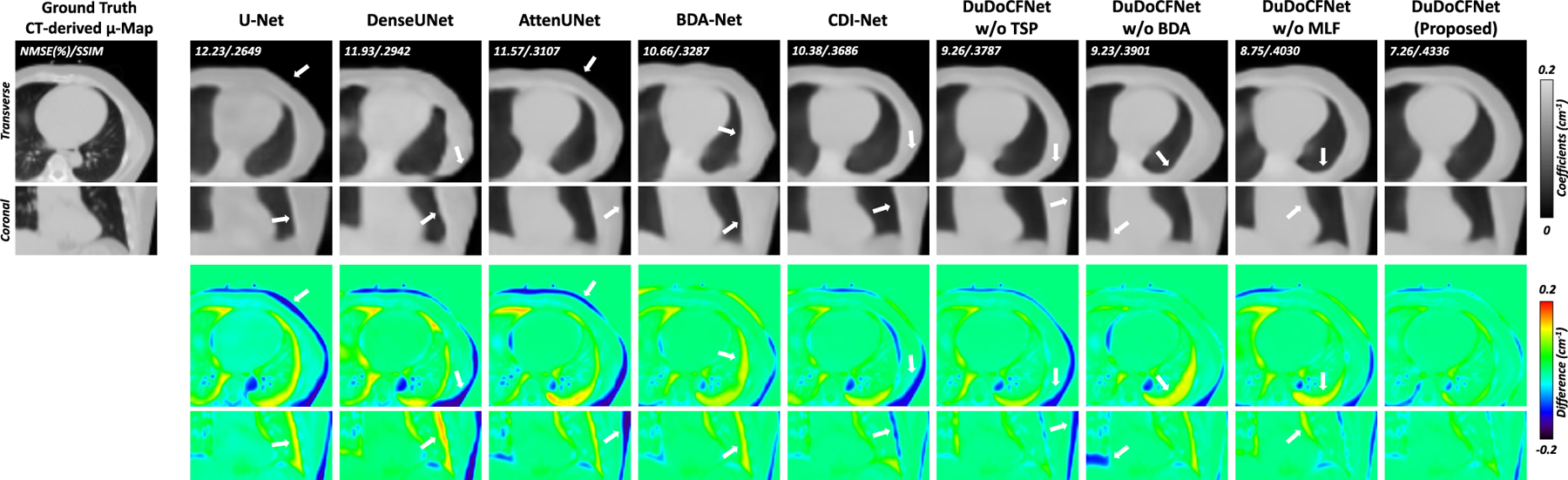
Predicted μ-maps (unit: cm^−1^) with error maps. White arrows denote the μ-map regions with inaccurate estimations. DuDoCFNet demonstrates the most accurate boundary estimations. NMSE and SSIM between the predicted and ground-truth μ-maps are annotated.

Fig. 7 shows the predicted μ-maps. The indirect approaches using U-Net, DenseUNet, and AttenUNet exhibit inaccurate estimations of μ-map boundaries. BDA-Net estimates a more accurate μ-map than AttenUNet, proving that the boundary enhancement mechanism (Fig. 4) improves the prediction accuracy. The predicted μ-map by DuDoCFNet is more consistent with ground truth compared to CDI-Net and the ablation groups. This demonstrates that the progressive learning strategies and the MLF mechanism in DuDoCFNet improve prediction accuracy. Table II illustrates the quantitative evaluations of the predicted μ-maps. BDA-Net generates more accurate μ-maps than other indirect methods but uses fewer parameters. The light-weighted DuDoCFNet predicts the most accurate μ-maps among all the testing groups and has fewer parameters than CDI-Net (NMSE, 11.43% vs 12.19%, p*<*0.001).

### C. Reconstructed AC SPECT Images

The predicted projections and μ-maps are then incorporated into the Maximum-Likelihood Expectation-Maximization reconstruction (ML-EM, 30 iterations) to produce AC SPECT images. The reconstruction group DuDoSS refers to reconstructing images using sequentially predicted full-view and denoised projections by DuDoSS [16] and predicted μ-map by AttenUNet [27], as the integration of the previous state-of-the-art methods.

**Fig. 8.**
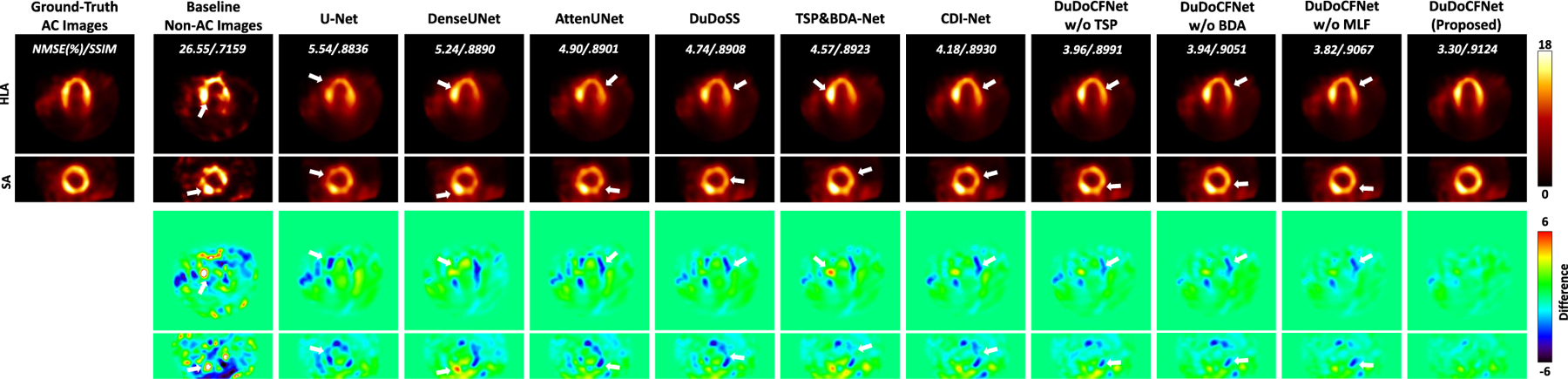
Reconstructed AC SPECT images using predicted projections and μ-maps presented in horizontal long axis (HLA) and short axis (SA) views. White arrows denote the image regions with inaccurate reconstructions. DuDoCFNet outputs the most accurate AC images. NMSE and SSIM between predicted and ground-truth images are annotated.

TSP&BDA-Net refers to the AC images reconstructed using predicted projections from TSP-Net and predicted μ-maps from BDA-Net. Fig. 8 shows the AC SPECT images. TSP&BDA-Net outputs more accurate AC images than existing single-task learning methods. The AC images generated by DuDoCFNet are more consistent with the ground truth, compared to single-task learning methods, CDI-Net, and the ablation groups. Table III lists the voxel-wise quantitative evaluations of the AC images. TSP&BDA-Net outputs more accurate results than existing single-task learning methods. In addition, DuDoCFNet produces more accurate AC images than the other testing methods and the ablation groups (NMSE, 4.34% vs 5.26%, p*<*0.001). This proves that DuDoCFNet significantly improves the final AC reconstruction accuracy.

**Fig. 9.**
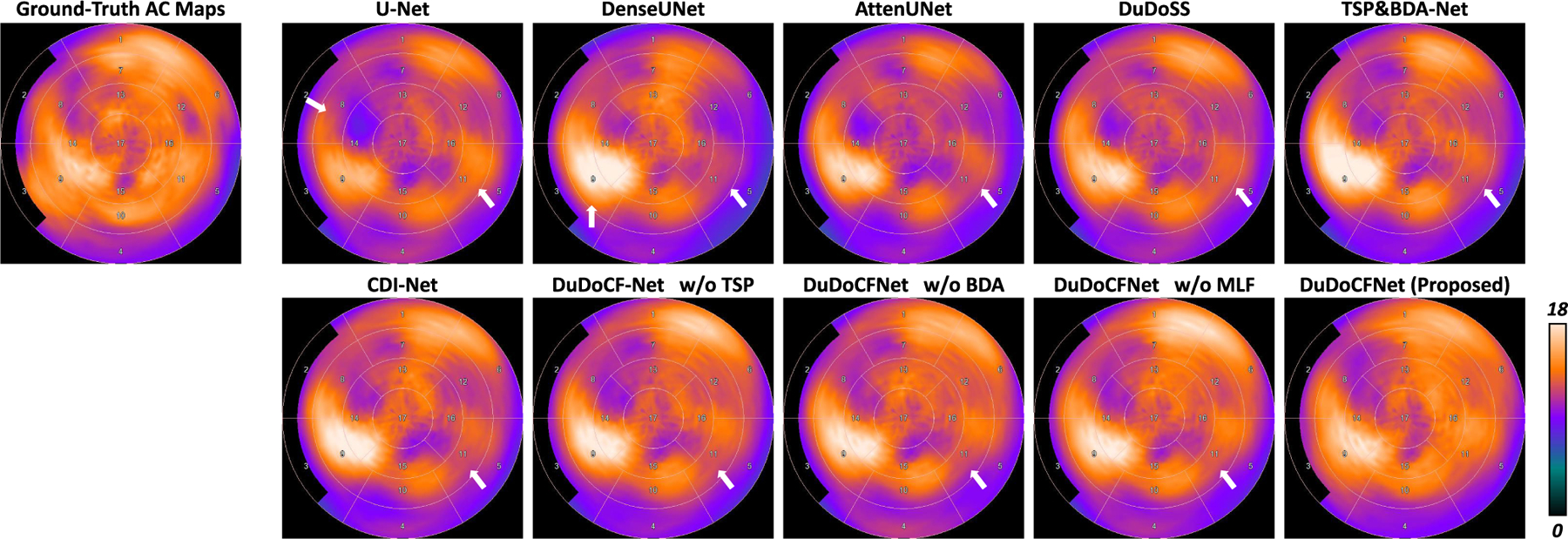
Standard 17-segment polar maps of the AC SPECT images. White arrows denote the segment regions with over- or under-estimated intensities. The polar map by DuDoCFNet is the most consistent with ground truth, compared to single-task, CDI-Net, and ablation study groups.

**Fig. 10.**
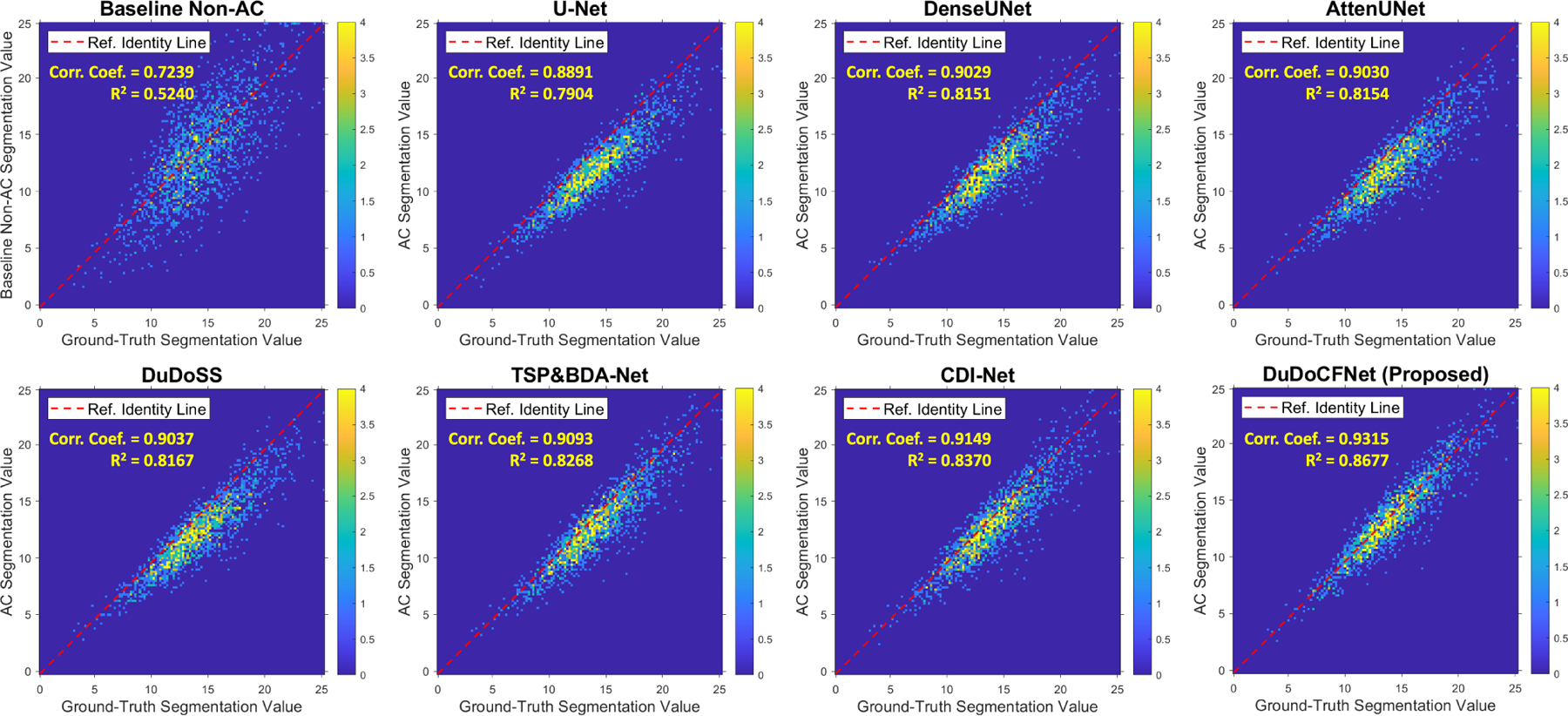
Correlation maps of segment values between polar maps of the ground-truth and estimated AC SPECT images. Correlation Coefficients (Corr. Coef.) and Coefficients of Determination (R^2^) are annotated.

The clinical standard 17-segment polar maps are generated from the AC SPECT images as shown in Fig. 9. The TSP&BDA-Net generates more accurate polar maps than existing single-task learning methods. In addition, the polar map by DuDoCFNet is the most consistent with the ground truth. Table IV presents the segment-wise quantification of the polar maps. The polar maps by DuDoCFNet demonstrate the lowest segment-wise errors (APE, 7.16% vs 9.03%, p*<*0.001) and the highest Correlation Coefficient (Corr. Coef.) among all testing groups. The correlation maps of the segment-wise values are shown in Fig. 10. DuDoCFNet shows the most concentrated point distributions and the highest Corr. Coef. This further proves that DuDoCFNet produces superior AC reconstruction results. The polar map patterns by DuDoCFNet are highly consistent with the clinical ground truth, which could largely enhance the SPECT imaging accuracy and thus improve the clinical diagnosis of coronary artery diseases.

### D. Impact of Iterations and Low-Dose Levels

**Fig. 11.**
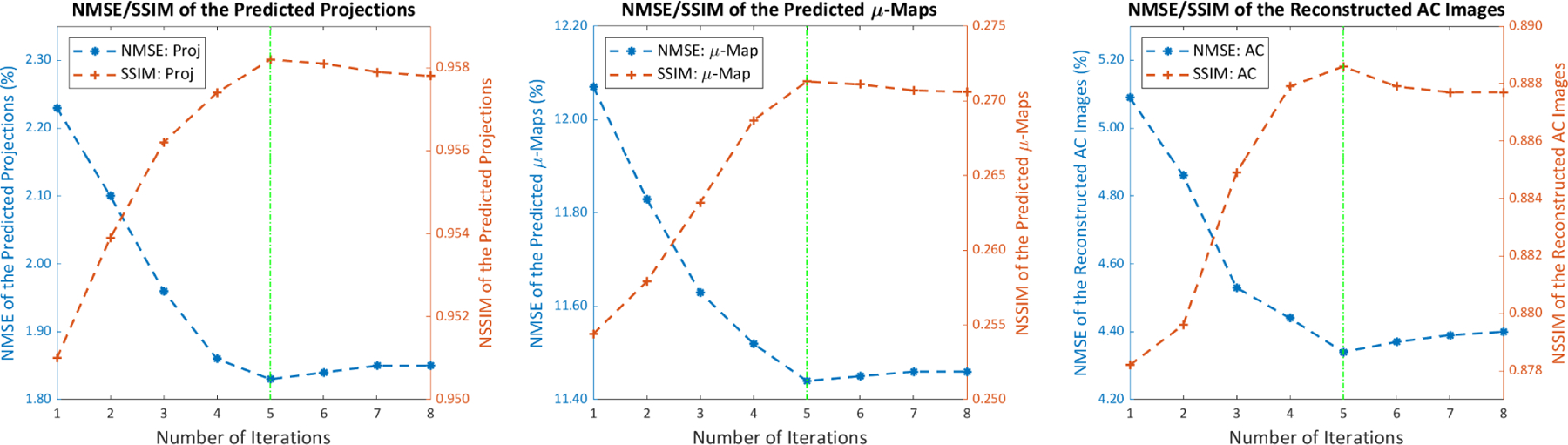
Quantitative evaluations of the predicted projections, μ-maps, and SPECT images by DuDoCFNet with number of iterations ranging from 1 to 8. DuDoCFNet’s performance improves as the number of iterations increases from 1 to 5. The network shows convergence over 5 iterations, and the prediction accuracy gradually decreases due to overfitting.

We further evaluated the performance of DuDoCFNet with different numbers of iterations as shown in Fig. 11. It can be observed that DuDoCFNet’s performance improves as the number of iterations increases from 1 to 5. However, after the number of iterations reaches 5, the network shows convergence, and the prediction accuracy gradually decreases due to overfitting. The possible reason is that, as proved by Kipf et al. [66], augmenting a deep neural network with additional layers can potentially lead to performance degradation due to overfitting, especially when the network’s depth exceeds what is necessary for the specific task and dataset. The optimal number of iterations is 5 for the dataset size employed in our study. However, higher optimal number of iterations and improved performance are expected with larger dataset, which can be validated in further down-stream clinical studies.

**Fig. 12.**
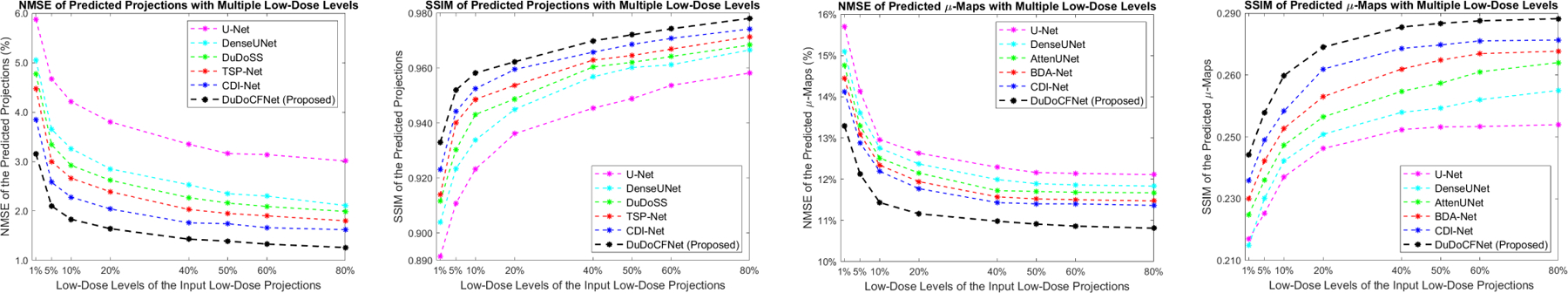
Quantitative evaluations of multiple groups over 8 datasets with low-dose levels varying from 1% to 80% using NMSE and SSIM.

In addition, we generated additional 7 datasets with varying LD levels ranging from 1% to 80%. These datasets were used to evaluate DuDoCFNet and existing methods under different LD levels as shown in Fig. 12. It can be observed that DuDoCFNet exhibits consistently superior accuracy in estimating projections and μ-maps under various dose settings.

### E. Impact of Image Padding

**Fig. 13.**
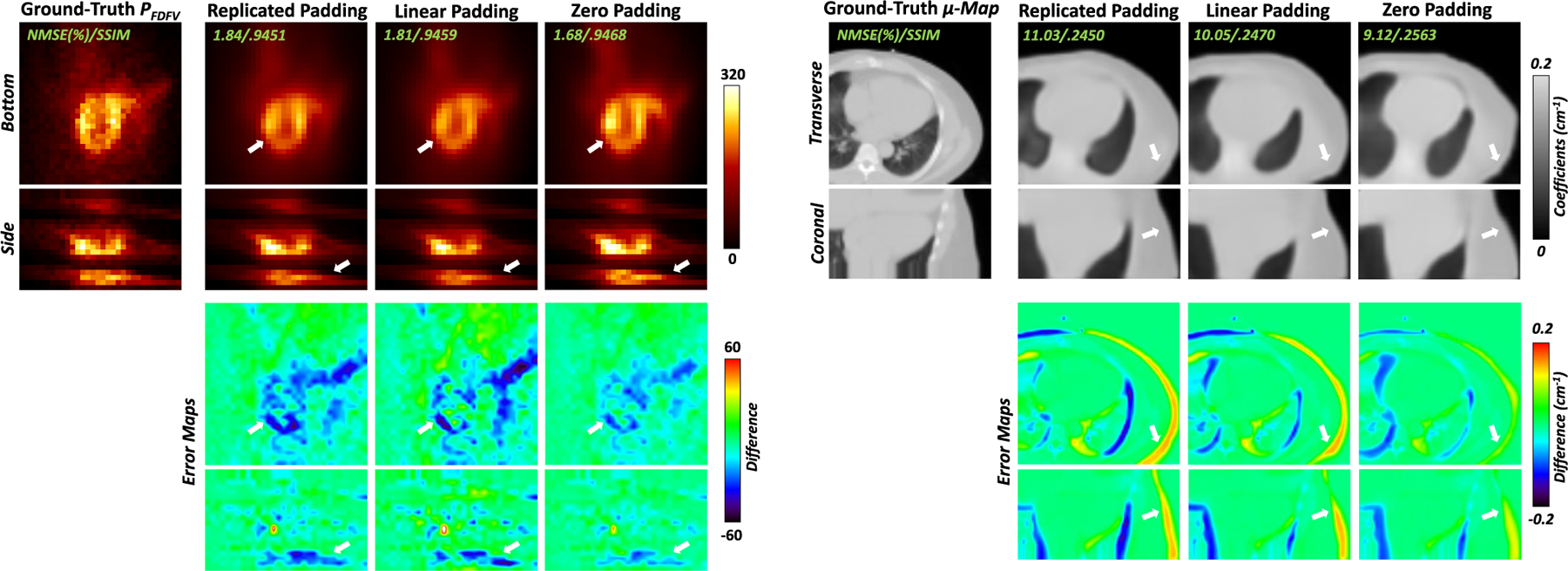
Visualizations of the predicted projections and μ-maps by DuDoCFNet using datasets with replicated padding, linear padding, or zero padding. White arrows denote the prediction inconsistency in the predicted projections and μ-maps.

We further investigated how the padding mechanism of LV projections affect DuDoCFNet’s prediction accuracy. We generated two additional datasets in which the LV projections are processed using replicated padding or linear padding. Then, we tested the performance of DuDoCFNet using the two newly generated datasets, and compared the performance with that using the default zero-padding dataset. Table V presents the quantitative evaluations of DuDoCFNet in predicting projections and μ-maps using datasets with replicated, linear, or zero padding. It can be observed that the Zero Padding group generates quantitatively more accurate FDFV projections (1.83% vs 2.00% or 2.02%, NMSE, p *<* 0.001) and μ-maps (11.43% vs 11.82% or 12.13%, p *<* 0.001) compared to the Replicated Padding and Linear Padding groups. Fig. 13 shows the predicted projections and μ-maps by DuDoCFNet using datasets with replicated, linear, or zero padding. It can be observed that the Zero Padding group predicts more consistent FDFV projections and μ-maps with ground truths, compared to the Replicated Padding and Linear Padding groups. Thus, according to the experimental results, the default zero padding mechanism shows better performance in predicting projections and μ-maps than replicated or linear padding.

### F. Investigations of Multiple Limited-View Configurations

**Fig. 14.**
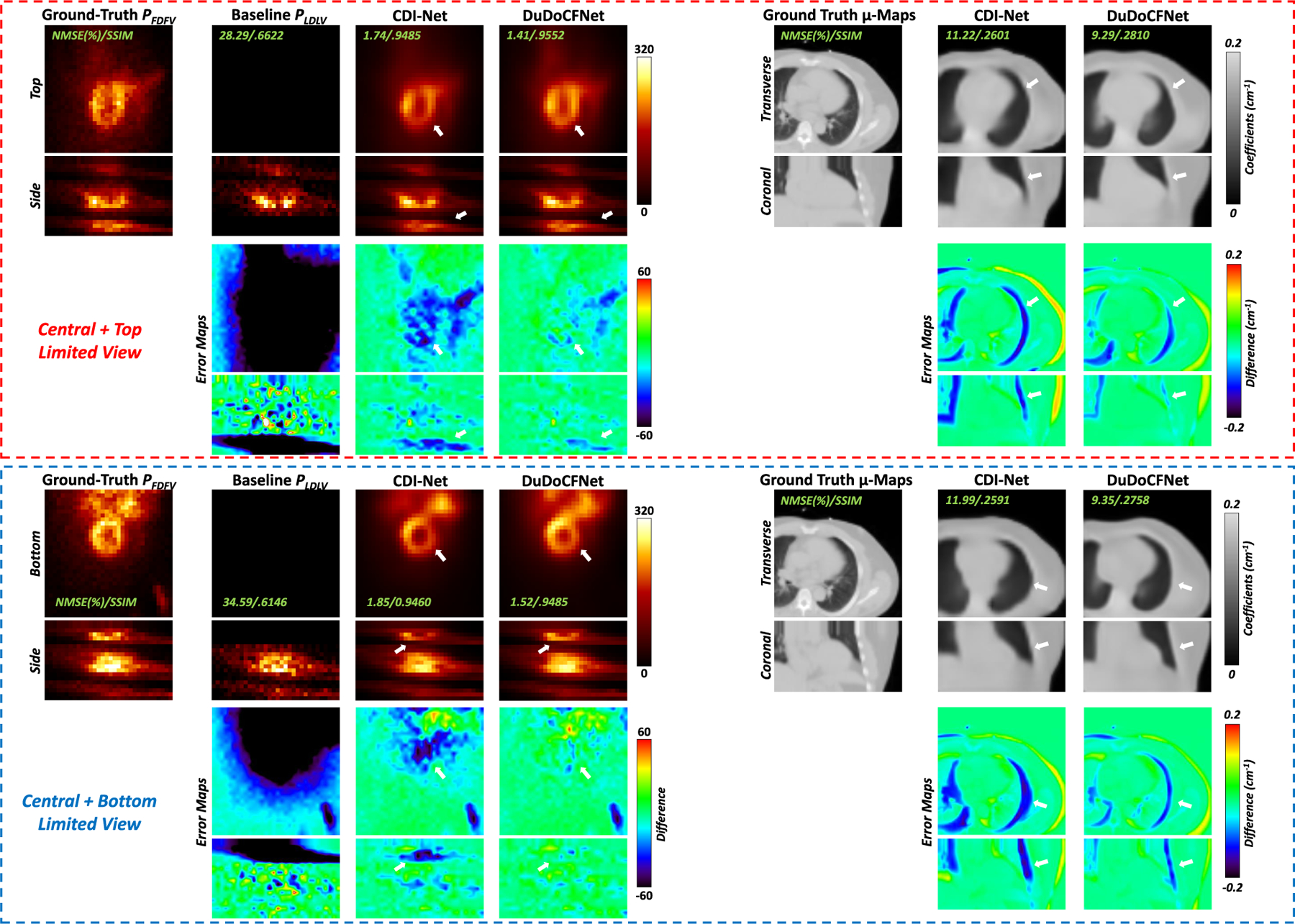
Visualizations of DuDoCFNet’s performance on two additional limited-view (LV) angle arrangement. The predicted projections and μ-maps using the Central + Top LV dataset are shown in the top red dash box. The predicted projections and μ-maps using the Central + Bottom LV dataset are shown in the bottom blue dash box. The while arrows denote the inconsistency of the predicted images.

In order to test DuDoCFNet’s performance on different LV arrangements, we generated another two datasets with different LV angle arrangement modes: (1) including the central 9 detectors and the top 5 detectors; (2) including the central 9 detectors and the bottom 5 detectors. Under the two additional LV datasets, we tested DuDoCFNet’s performance and compared that with the performance of the previous state-of-the-art simultaneous learning technique in cardiac SPECT, CDI-Net. Table VI presents the quantitative evaluations of the predicted FDFV projections and μ-maps by DuDoCFNet and CDI-Net using the two additional datasets. It can be observed in the table below that DuDoCFNet predicts quantitatively more accurate FDFV projections (1.29% vs 1.52%, 1.63 vs 1.84%, NMSE, p *<* 0.001) and μ-maps (11.13% vs 11.82%, 11.35% vs 11.85%, NMSE, p *<* 0.001) compared to CDI-Net, on both LV datasets. Fig. 14 shows the predicted FDFV projections and μ-maps on the two additional datasets. It can be observed that DuDoCFNet generates more consistent FDFV projections and μ-maps with the ground truths compared to CDI-Net, on both additional LV datasets. Thus, according to the experimental results, DuDoCFNet shows consistently superior performance on different LV arrangement modes.

## IV. Discussion

In this work, we propose DuDoCFNet, a multi-task learning method, for simultaneous LD denoising, LV reconstruction, and CT-free μ-map generation of cardiac SPECT. Specifically, DuDoCFNet employs a dual-domain cascaded framework that enables cross-domain feature fusion. In DuDoCFNet, projection-domain and image-domain networks are concatenated through forward and backward projections to enable data flow across the two distinct domains. This framework effectively fuses the cross-domain and cross-modality information to enhance the prediction accuracy of each individual task. The MLF mechanism effectively connects and fuses the cross-modality image features at different spatial dimensions. The embedded CDF modules are utilized to adaptively adjust the channel-wise weights to enhance the cross-modality feature fusion. The TSP-Net and BDA-Net within DuDoCFNet share similar design motivations that utilize two-stage architectures for progressive coarse-to-fine estimations of projections or μ-maps. Specifically, TSP-Net employs a U-Net-like structure to restore general structures and a non-downsampling module to recover finer details of the LA and LD projections. BDA-Net employs a shared encoder to predict a coarse μ-map and the boundary, followed by a spatial co-attention module to generate a refined μ-map with enhanced boundary accuracy. Specifically, the overall dual-domain structure of DuDoCFNet is designed to generate SPECT projections in the projection domain and generate μ-maps in the image domain, respectively. The FP and BP operators are utilized for linearly transforming the image matrix, thus guaranteeing consistent image dimensions after cross-domain connections. Due to the discrepancy of the cross-domain and cross-modality image features, the MLF mechanism is employed as an adaptive data fusion manner for dynamic integration of these diverse image features. The first stage of TSP-Net, a U-Net-like structure, is intended for recovering the general structure of the input LV projection by the feature extraction capabilities of the downsampling-upsampling backbones. The second stage of TSP-Net applies a non-downsampling convolutional module, intended for restoring finer image details by the identical-dimension convolution structures. In contrast, the first stage of BDA-Net is designed to estimate the overall coarse structure and more importantly the boundary of the μ-maps that are pre-requisites for the boundary restoration in the next stage. The second stage of BDA-Net first applies a CDF module to adaptively fuse the predicted coarse μ-maps and boundaries and also enhance the network’s spatial attention on the μ-map boundaries. A subsequent CNN module is then employed to encode the fused general and boundary information, thus generating a more consistent μ-map with improved boundary consistency.

The advantages of the two-stage progressive learning strategies are dividing and distributing an otherwise challenging task to two end-to-end connected specialized modules. Each module is specifically designed to concentrate on one aspect of the task, thereby enhancing not only the predictive accuracy of each individual component but also improving the overall performance of the system. The main disadvantage of the two-stage progressive learning strategies is the relatively specialized and complicated network structures. The module of each stage must be customized to align with the specific context and demands of each subtask, in order to reach the optimal overall performance. Thus, this approach might lack a “one-size-fits-all” framework suitable for all the complex tasks. Moreover, the complicated structure of the two-stage network typically requires additional computational resources. To minimize the computational costs, DuDoCFNet is designed to be super light-weighted by reducing the number of channels in its convolutional layers. To summarize briefly, this work features the first dual-domain approach for multi-task simultaneous learning in nuclear medicine. The methodology aspects demand a considerable amount of technical innovations to realize the novel multi-task combination and improvement. Specifically, our contributions in this work include: 1) we proposed the cross-domain and cross-modality data fusion framework, which can effectively fuse the image features across multiple domains and imaging modalities and thus improve the network performance in each individual domain; 2) we developed a MLF mechanism and a CDF module to enable more accurate data fusion across multiple domains and in different spatial dimensions; 3) we designed the TSP-Net that employs the “two-stage progressive estimation” principle for simultaneous denoising and limited-view restoration of SPECT projections; 4) we designed the BDA-Net that utilizes the “coarse-to-fine boundary enhancement” principle for estimating more accurate μ-maps with enhanced boundary consistency.

Our experiments based on clinical data demonstrated that DuDoCFNet predicts more accurate projections and μ-maps than existing single-task learning methods. It can be noticed in Fig. 6 that previous deep learning methods under-estimate the projection intensities especially at the cardiac regions, which was also noted in a previous study [16]. As a possible explanation, the under-estimations might be attributed to the initial input values in the areas with missing projection information, which are zeros and significantly lower than surrounding areas. Consequently, neural networks tend to output relatively lower values in these specific areas with significantly lower input values. This phenomenon could be affected by imaging modalities, data properties, network architectures, etc. Our proposed DuDoCFNet can largely improve the under-estimations as shown in Fig. 6. Further studies can be conducted to explore and address the projection under-estimation issues in the LV reconstruction applications. Additionally, DuDoCFNet has fewer network parameters but performs better than the previous multi-task learning framework CDI-Net, as indicated in Tables I and II. Ablation studies of DuDoCFNet further validate the impact of the proposed two-stage progressive coarse-to-fine estimation strategies and MLF mechanism on improving network performance. Moreover, we demonstrated that DuDoCFNet’s performance, based on the dataset in this study, improves as the number of iterations increases and converges at 5 iterations due to overfitting, as illustrated in Fig. 11, higher optimal number of iterations and improved performance could be obtained with larger datasets, which will be validated in our future studies. Notably, even with only 1 iteration, DuDoCFNet still outperforms TSP-Net in predicting projections (2.23% vs 2.66%, p *<* 0.001) and BDA-Net in predicting μ-maps (12.07% vs 12.34%, p *<* 0.001). This further proves that the simultaneous learning framework of DuDoCFNet improves the prediction accuracy of each interrelated task. In addition, we demonstrated that DuDoCFNet consistently exhibits superior performance under varying LD levels from 1% to 80%, as presented in Fig. 12. Then, we evaluated the reconstruction accuracy of DuDoCFNet in terms of the reconstructed AC SPECT images and the clinical standard 17-segment polar maps. The polar map patterns by DuDoCFNet are highly consistent with the ground truth, which could largely enhance the SPECT imaging accuracy and thus improve the clinical diagnostic capabilities for coronary artery diseases. Furthermore, we investigated the impact of image padding mechanism on DuDoCFNet’s prediction accuracy and proved that the zero padding we used generated the optimal results. A possible reason of this result is associated with the unique configurations of the GE NM/CT 570c scanners involved in our study, as presented in Fig. 1. The GE 570c scanner includes 19 detectors in total with pinhole collimators, with 5, 9, 5 placed on the top, central, and bottom columns. The input LV projections include the central 9 detectors in our study. However, due to the irregular shape of the scanner and pinhole collimation, the detectors on the top and bottom columns are not spatially aligned with the detectors on the central column. Specifically, the angular arrangements of the top/bottom columns are different from that of the central column, in terms of angular intervals and detectors’ positions on the z-axis. Therefore, using replicated or linear padding will introduce projection information that do not match the true data of the top and bottom detectors, leading to a deviation from the ground-truth FV projections and potentially reducing prediction accuracy. Finally, we investigated DuDoCFNet’s performance under multiple LV configurations and proved that DuDoCFNet generated consistently accurate results on different LV arrangement modes.

Our current work also has some potential limitations. First, DuDoCFNet employs an iterative framework that consists of cascaded TSP-Nets and BDA-Nets. Consequently, the computational costs associated with DuDoCFNet are relatively high compared to the single-task learning methods such as DenseUNet [59] and AttenUNet [60], particularly when multiple iterations are employed. However, even with just 1 iteration, DuDoCFNet still demonstrates promising performance. The inference time of DuDoCFNet is *<* 1s even with high iterations, which is a reasonable speed for clinical practice. Second, although DuDoCFNet can generate voxel-wise accurate μ-maps with enhanced boundary consistency, finer details of spine bone structures still cannot be well estimated as shown in Fig. 7. Due to the limited field-of-view of the dedicated cardiac SPECT scanner in this study, the input SPECT information is inaccurate outside the cardiac regions, for example, the spine regions. Thus, predicting accurate spine bone structures with incomplete emission information as input is quite challenging now [27]. The investigation of advanced deep learning techniques for estimating μ-maps with finer details in dedicated SPECT scanner is also an interesting research topic. Third, in this study, DuDoCFNet is only tested using clinical data from cardiac SPECT-CT scanners. More validations of DuDoCFNet with diverse datasets from various tracers, scanners, organs, and imaging modalities could be conducted to comprehensively assess DuDoCFNet’s capabilities under different clinical scenarios. Fourth, the primary emphasis of this study lies in the methodology development and validation of DuDoCFNet. While we have generated and analyzed the standard 17-segment polar maps using clinical analysis tools, the clinical validation of DuDoCFNet remains insufficient and warrants further investigation and evaluation before being employed in clinical practice. Last, the applications of current deep learning-based reconstruction models, including our proposed DoDuCFNet, are typically limited to some specific hardware configurations. As a result, the well-trained models cannot be easily deployed into different clinics due to the distinct machine hardware architectures. Thus, integrating machine parameters into the model as another input channel would facilitate broader and more universal clinical applications of current deep learning algorithms.

There are some factors worth attention in cardiac SPECT including motion correction and scatter correction. Motion correction methods for this GE 530c/570c dedicated scanner were investigated by several studies [67], [68], [69], [70]. The post-reconstruction method can also be employed for reducing the respiratory and cardiac motions [71]. These motion correction methods can be investigated and directly integrated into our DuDoCFNet framework in our future clinical studies to further improve the clinical reconstruction accuracy. For the proposed dedicated cardiac scanners with cadmium zinc telluride (CZT) detectors, conventional scatter correction methods such as the triple energy window (TEW) or dual energy window (DEW) [72] could not be directly applied due to the CZT tailing effect. Approaches have been developed to account for the tailing effect in corrections for scatter and crosstalk [73], [74], [75]. Such methods can be directly integrated into our DuDoCFNet framework in the future studies.

Moreover, the ML-EM algorithm is derived based on Poisson distributed data [76], [77], but the deep-learning-based synthetic projections are not strictly Poisson distributed. The ML-EM algorithm has previously been successfully employed even when the data is not Poisson distributed [16], [78], [79]. In addition, out proposed DuDoCFNet framework is “reconstruction algorithm neutral” and not specific to ML-EM. Alternative algorithms like Maximum a Posteriori (MAP) [80] and Penalized Weighted Least-Squared (PWLS) [81] could also be applied to replace ML-EM in our study depending on users’ specific requirements and preferences.

Our work suggests some promising directions for future studies. First, DuDoCFNet indicates the significance of cross-modality feature fusion in enhancing prediction accuracy. As indicated in this study, the complementary information from emission and anatomical images enables more comprehensive feature representations and thus higher prediction accuracy. Hence, leveraging multi-modality features can potentially improve the performance of many medical imaging applications such as segmentation, registration, etc. Second, the multi-stage progressive learning strategy offers a viable solution for many complicated applications. As shown in our study, separating a complex learning task into several sections and utilizing specialized network modules for each individual section can enhance the overall performance. Third, the ML-EM algorithm is employed in our study for post reconstruction to keep aligned with the clinical practice. However, additional image reconstruction algorithms, such as the maximum a posteriori reconstruction with TV regularization [82], can be explored in our future studies to further enhance the reconstruction accuracy. Last, this study showed that simultaneous learning of interrelated tasks can largely enhance the performance of each task. Therefore, further investigation is warranted for interrelated tasks such as denoising [10] and registration [56], [83], [84], [85], which can be learned simultaneously.

## V. Conclusion

In conclusion, we propose DuDoCFNet for simultaneous LD denoising, LV reconstruction, and CT-free AC of cardiac SPECT. DuDoCFNet enables accurate and accelerated AC SPECT imaging while reducing hardware expenses and minimizing radiation exposure. The cascaded framework fuses the cross-domain and cross-modality image features for simultaneous learning. The two-stage progressive learning strategies improve estimation accuracy in both projection and image domains. Experiments with clinical data exhibit the superior performance of DuDoCFNet in predicting projections, generating μ-maps, and AC reconstructions, compared to existing single- or multi-task learning methods. The clinical segment-wise evaluations using standard polar maps demonstrate that the AC reconstructions by DuDoCFNet are highly consistent with the clinical ground truth. This could largely enhance the SPECT MPI imaging accuracy and further improve the clinical diagnostic capabilities for coronary artery diseases.

## Figures and Tables

**TABLE I:** Quantitative Evaluations of Predicted Projections on 250 Testing Cases Using NMSE, SSIM, and PSNR. The Numbers of Network Parameters (Param, Unit: Million) Are Indicated in the Last Column. The Best Results Are Marked in **BOLD**

Methods	NMSE (%)	SSIM	PSNR	P-values^a^	Param (m)
Baseline LDLV	54.35 ± 2.34	.492 ± .025	19.14 ± 1.69	< 0.001	–
LDFV Projection	8.64 ± 2.38	.845 ± .048	29.36 ± 2.66	< 0.001	–
U-Net [14]	4.21 ± 1.41	.923 ± .024	30.45 ± 1.46	< 0.001	1.36
DenseUNet [59]	3.26 ± 1.02	.938 ± .015	31.55 ± 1.50	< 0.001	1.43
AttenUNet [60]	3.11 ± 1.01	.943 ± .015	31.77 ± 1.45	< 0.001	1.54
DuDoSS [16]	2.92 ± 0.96	.943 ± .016	32.04 ± 1.49	< 0.001	3.09
End2End-Net [17]	2.91 ± 0.85	.944 ± .015	32.10 ± 1.50	< 0.001	2.88
TSP-Net [Fig. 3]	2.66 ± 0.81	.949 ± .015	32.43 ± 1.65	< 0.001	1.01
CDI-Net [57]	2.28 ± 0.74	.950 ± .015	33.12 ± 1.66	< 0.001	3.11×N^b^
DuDoCFNet w/o TSP	2.15 ± 0.64	.955 ± .013	33.34 ± 1.73	< 0.001	1.42×N
DuDoCFNet w/o BDA	2.12 ± 0.65	.954 ± .013	33.42 ± 1.68	< 0.001	1.52×N
DuDoCFNet w/o MLF	2.09 ± 0.68	.955 ± .013	33.51 ± 1.68	< 0.001	1.76×N
**DuDoCFNet (Proposed)**	**1.83 ± 0.57**	**.958 ± .013**	**34.07 ± 1.68**	**–**	**1.77×N**

a P-values of paired t-tests on NMSE between the current testing group and DuDoCFNet (Proposed).

b Number of iterations of the deep learning framework with a default of 5.

**TABLE II:** Quantitative Evaluations of Predicted μ-Maps on 250 Testing Cases Using NMSE, SSIM, and PSNR. The Numbers of Network Parameters (Param, Unit: Million) Are Indicated in the Last Column. The Best Results Are Marked in **BOLD**

Methods	NMSE (%)	SSIM	PSNR	P-values^a^	Param (m)
U-Net [14]	12.96 ± 4.90	.237 ± .047	17.26 ± 1.82	< 0.001	1.36
DenseUNet [59]	12.75 ± 5.13	.242 ± .048	17.30 ± 1.83	< 0.001	1.43
AttenUNet [60]	12.51 ± 4.33	.247 ± .050	17.33 ± 1.82	< 0.001	1.54
BDA-Net [Fig. 4]	12.34 ± 4.46	.253 ± .048	17.41 ± 1.84	< 0.001	0.77
CDI-Net [57]	12.19 ± 5.04	.258 ± .047	17.51 ± 1.89	< 0.001	3.11×N^b^
DuDoCFNet w/o TSP	11.91 ± 4.77	.264 ± .057	17.60 ± 1.84	< 0.001	1.42×N
DuDoCFNet w/o BDA	11.97 ± 4.90	.263 ± .057	17.58 ± 1.91	< 0.001	1.52×N
DuDoCFNet w/o MLF	11.89 ± 4.72	.265 ± .055	17.60 ± 1.88	< 0.001	1.76×N
**DuDoCFNet (Proposed)**	**11.43 ± 4.67**	**.270 ± .058**	**17.83 ± 1.85**	**–**	**1.77×N**

a P-values of paired t-tests on NMSE between the current testing group and DuDoCFNet (Proposed).

b Number of iterations of the deep learning framework with a default of 5.

**TABLE III:** Quantitative Evaluations of Reconstructed AC SPECT Images Using Predicted Projections and μ-Maps on 250 Testing Cases. The Best Results Are Marked in **BOLD**

Methods	NMSE(%)	SSIM	PSNR	P-values^a^
Baseline Non-AC	35.17 ± 10.35	.665 ± .034	24.10 ± 1.82	< 0.001
U-Net [14]	6.55 ± 1.88	.863 ± .021	31.44 ± 1.59	< 0.001
DenseUNet [59]	6.27 ± 1.80	.864 ± .020	31.62 ± 1.56	< 0.001
AttenUNet [60]	6.18 ± 1.84	.864 ± .021	31.63 ± 1.56	< 0.001
DuDoSS [16]	6.12 ± 1.83	.865 ± .021	31.67 ± 1.56	< 0.001
TSP&BDA-Net [Fig. 3, 4]	5.38 ± 1.73	.875 ± .021	32.26 ± 1.66	< 0.001
CDI-Net [57]	5.26 ± 1.61	.876 ± .021	32.33 ± 1.65	< 0.001
DuDoCFNet w/o TSP	4.87 ± 1.43	.880 ± .020	32.67 ± 1.63	< 0.001
DuDoCFNet w/o BDA	4.78 ± 1.40	.882 ± .019	32.73 ± 1.61	< 0.001
DuDoCFNet w/o MLF	4.75 ± 1.32	.882 ± .019	32.76 ± 1.60	< 0.001
**DuDoCFNet (Proposed)**	**4.34 ± 1.32**	**.889 ± .019**	**33.07 ± 1.67**	**–**

a P-values of paired t-tests on NMSE between the current testing group and DuDoCFNet (Proposed).

**TABLE IV:** Segment-Wise Quantitative Evaluations of Polar Maps on 100 Testing Cases Using APE, Correlation Coefficient (Corr. Coef.), and Coefficient of Determination (R^2^). The Best Results Are Marked in **BOLD**

Methods	APE(%)	P-values^a^	Corr. Coef.	R^2^
Baseline Non-AC	16.99 ± 13.11	< 0.001	0.7239	0.5240
U-Net [14]	13.63 ± 7.97	< 0.001	0.8891	0.7904
DenseUNet [59]	12.62 ± 8.00	< 0.001	0.9029	0.8151
AttenUNet [60]	12.52 ± 7.96	< 0.001	0.9030	0.8154
DuDoSS [16]	12.07 ± 7.92	< 0.001	0.9037	0.8167
TSP&BDA-Net [Fig. 3, 4]	9.13 ± 7.04	< 0.001	0.9093	0.8268
CDI-Net [57]	9.03 ± 7.14	< 0.001	0.9149	0.8370
DuDoCFNet w/o TSP	8.30 ± 6.84	< 0.001	0.9159	0.8388
DuDoCFNet w/o BDA	8.25 ± 6.27	< 0.001	0.9262	0.8579
DuDoCFNet w/o MLF	8.13 ± 6.10	< 0.001	0.9282	0.8615
**DuDoCFNet (Proposed)**	**7.16 ± 5.77**	**–**	**0.9315**	**0.8677**

a P-values of paired t-tests on APE between the current testing group and DuDoCFNet (Proposed)

**TABLE V:** Quantitative Evaluations of DuDoCFNet in Predicting Projections and μ-Maps Using Datasets With Replicated Padding, Linear Padding, or Zero Padding. The Best Performing Results Are Marked in **BOLD**

	Predicted FDFV projections			Predicted μ-maps		
	NMSE (%)	SSIM	PSNR	NMSE (%)	SSIM	PSNR
Replicated Padding	2.02 ± 0.73	.954 ± .014	33.68 ± 1.78	11.82 ± 4.67	.250 ± .052	17.62 ± 1.84
Linear Padding	2.00 ± 0.61	.956 ± .013	33.65 ± 1.79	12.13 ± 5.23	.244 ± .050	17.55 ± 1.89
**Zero Padding**	**1.83 ± 0.57**	**.958 ± .013**	**34.07 ± 1.68**	**11.43 ± 4.67**	**.270 ± .051**	**17.83 ± 1.85**

**TABLE VI:** Quantitative Evaluations of DuDoCFNet’s Performance on Two Additional Limited-View (LV) Angle Arrangements. The Best Performing Groups Are Marked in **BOLD**

	Predicted FDFV projections			Predicted μ-maps		
	NMSE (%)	SSIM	PSNR	NMSE (%)	SSIM	PSNR
*Additional LV dataset 1: Central + Top angles*						
Baseline LDLV	25.34 ± 3.64	.703 ± .038	22.50 ± 2.02	–		
CDI-Net [57]	1.52 ± 0.52	.961 ± .014	34.90 ± 2.16	11.82 ± 4.78	.268 ± .058	17.661 ± 1.88
**DuDoCFNet**	**1.29 ± 0.41**	**.963 ± .012**	**35.65 ± 2.08**	**11.13 ± 4.93**	**.279 ± .055**	**17.99 ± 1.86**
*Additional LV Dataset 2: Central + Bottom angles*						
Baseline LDLV	34.65 ± 4.12	.634 ± .038	21.12 ± 1.62	–		
CDI-Net [57]	1.84 ± 0.66	.959 ± .013	34.09 ± 1.65	11.85 ± 4.48	.266 ± .050	17.58 ± 1.86
**DuDoCFNet**	**1.63 ± 0.54**	**.961 ± .013**	**34.58 ± 1.74**	**11.35 ± 4.57**	**.271 ± .053**	**17.91 ± 1.87**
